# Efficacy of brolucizumab and ranibizumab in diabetic macular edema and neovascular age-related macular degeneration: insights from a case series

**DOI:** 10.1097/MS9.0000000000003363

**Published:** 2026-03-18

**Authors:** Billal Hossain, Sabrina Rahmatullah, Tanvir Ahmed

**Affiliations:** a Lieutenant Colonel, Consultant, Vitreo-Retinal Surgeon, Bangladesh Eye Hospital, Dhaka, Bangladesh; b Vitreo-Retinal and Phaco Surgeon, Bangladesh Eye Hospital, Dhaka, Bangladesh; c Vitreo-Retinal and Phaco Surgeon, Bangladesh Eye Hospital, Dhaka, Bangladesh

**Keywords:** brolucizumab, diabetic macular edema, neovascular age-related macular degeneration, ranibizumab

## Abstract

**Background:**

The effectiveness of various anti-vascular endothelial growth factors in treating patients with diabetic macular edema (DME) and neovascular age-related macular degeneration (nAMD) in Bangladesh lacks comprehensive information.

**Objective:**

We evaluated the efficacy of brolucizumab and ranibizumab and assessed the incidence of drug-related adverse events in nAMD and DME patients of Bangladesh.

**Methods:**

In this retrospective case series, 20 patients who had received either intravitreal brolucizumab (n = 10) or ranibizumab (n = 10) were included. Demographic and clinical characteristics, data on best-corrected visual acuity (BCVA), and optical coherence tomography (OCT) measurements, including macular volume (MV) and central subfield retinal thickness (CSRT), were recorded for each patient at baseline and follow-up visits. In addition, brolucizumab was compared with ranibizumab in terms of above mentioned parameters.

**Results:**

Both brolucizumab and ranibizumab induced changes in patient parameters, including BCVA, OCT measurements, and MV, relative to baseline values. At the 12-week follow-up, neither drug demonstrated statistically significant superiority over the other (*P* > 0.05 in all cases). However, over the course of treatment, the mean BCVA showed a significant improvement following the initiation of brolucizumab therapy (*P* = 0.042). In terms of OCT findings, the mean CSRT significantly decreased from 508.5 ± 101.1 µm at baseline to 241 ± 40.4 µm at week 12, while the mean MV was significantly reduced from 10.3 ± 1.0 mm^3^ to 8.2 ± 1.6 mm^3^ (*P* < 0.05).

**Conclusion:**

Intravitreal brolucizumab appeared to be an effective option for patients with nAMD and DME.

## Introduction

Diabetic macular edema (DME) and neovascular age-related macular degeneration (nAMD) are two significant causes of vision impairment and central vision loss among individuals worldwide^[^1-3^]^. Key factors that seem to have a dominant role in DME and nAMD are angiotensin II, prostaglandins and the vascular endothelial growth factor (VEGF) and a deficiency of anti-inflammatory bioactive lipids which may initiate the onset and progression of DME and AMD^[^4,5^]^.

**HIGHLIGHTS**

- Diabetic macular edema (DME) and age-related macular degeneration (AMD) are leading causes of vision impairment and central vision loss globally.
- Various treatment strategies have been explored for DME and AMD, with intravitreal anti-vascular endothelial growth factors (anti-VEGFs) emerging as both safe and effective.
- Brolucizumab, a novel anti-VEGF agent, has shown to enhance best corrected visual acuity (BCVA) and reduce retinal thickness in patients with DME and AMD.
- Brolucizumab offers comparable effectiveness and safety to other anti-VEGF therapies.

A variety of approaches to the treatment of DME and AMD have been attempted, with a variable degree of success, of which intravitreal Anti-VEGF therapies have proven to be effective^[^6,7^]^. Despite the widespread use of effective intravitreal anti-VEGF therapy for AMD and DME, significant obstacles to receiving care remain. Successful execution of the procedure requires expertise. Moreover, receiving the treatment necessitates prior consultation. In many settings, the designated intervention sites are not readily accessible, requiring patients to travel, which incurs additional costs. Furthermore, the presence of a caregiver during and after the procedure is essential, imposing a significant burden on both the personal and professional lives of patients and their caregivers^[^5,8-11^]^. Furthermore, patients may need extra healthcare visits to address other health conditions since individuals with DME and AMD are more likely to experience comorbidities unrelated to their disease and treatment when compared to non-diabetic patients with AMD and healthy controls, respectively^[^12,13^]^.

In response to these challenges and with the objective of sustaining visual acuity improvements while effectively managing fluid retention, several anti-VEGF agents have been approved and widely utilized worldwide, including brolucizumab, aflibercept, ranibizumab, and faricimab. Additionally, bevacizumab has been used off-label and extensively studied in clinical settings globally. Ranibizumab is a 48 kDa humanized recombinant antibody fragment (Fab) that neutralizes all active forms of vascular endothelial growth factor A (VEGF-A). In contrast, brolucizumab is a 26 kDa humanized single-chain antibody fragment composed of the variable domains of a monoclonal antibody connected by a flexible peptide linker, enabling it to bind all VEGF-A isoforms^[^14^]^. Among these agents, it was thought that brolucizumab has certain advantage over others for the treatment of both age-related macular degeneration (AMD) and DME. Due to its smaller molecular weight (26 kDa), brolucizumab facilitates higher molar dosing, leading to an extended duration of action, enhanced tissue penetrability, and a reduced injection frequency. These characteristics contribute to a lower treatment burden for patients with AMD and DME compared to earlier anti-VEGF therapies.^[^5,15^]^. In the Phase III HAWK and HARRIER trials, brolucizumab 6 mg showed similar improvements in best-corrected visual acuity (BCVA) compared to aflibercept, with better anatomical outcomes in patients with nAMD. Over 50% of patients were able to maintain a 12-week dosing schedule after the initial loading phase through Week 48^[^15^]^. Similarly, the KESTREL and KITE trials for DME demonstrated that after five initial loading doses every 6 weeks, brolucizumab could be continued on a 12-week interval, with more than half of patients maintaining this schedule through Week 52^[^16^]^. However, brolucizumab has been associated with adverse events of intraocular inflammation (IOI), retinal vasculitis, and retinal vascular occlusion^[^17,18^]^.

Despite these promising results, there is a gap in the real-world data on the effectiveness of various anti-VEGF treatments for vision loss in patients with nAMD and DME, especially in Bangladesh. Therefore, in this case series we aimed to assess visual acuity, retinal thickness, macular volume, and any adverse events in 20 patients (20 eyes) with DME and AMD who have undergone brolucizumab and ranibizumab treatment in Bangladesh. By evaluating these parameters, we aim to gain valuable insights into the effectiveness and adverse events of these two anti VEGFs agents in this specific patient population, ultimately contributing to a better understanding of its role in managing DME and AMD patients in Bangladesh.

## Method

### Patient selection and ethics

This retrospective case series analyzed the medical records of 20 patients diagnosed with nAMD or DME from September 2023 to July 2024. Patients aged 18 years or older who required treatment with anti-VEGF agents were included in the study. The cohort comprised of both treatment-naïve individuals and those with therapy-refractory conditions. Therapy-refractory cases were defined as patients with persistent nAMD or DME despite undergoing at least 6 months of anti-VEGF therapy with bevacizumab, ranibizumab, and/or aflibercept.

A total of 20 eyes were evaluated, with 10 eyes treated with intravitreal ranibizumab and the other 10 receiving intravitreal brolucizumab. Participants were selected through convenience sampling, taking into consideration loss to follow-up and missing data within the available medical records.

The study was reviewed and approved by the ethical review committee of Public Health Foundation Bangladesh (PHFBD-ERC-SF04/2024). The research was conducted in adherence to the principles of the Declaration of Helsinki. Informed consent was not required for this study given its retrospective design. This case series has been reported in line with the PROCESS Guideline^[^19^]^.

### Study procedure and measurements

Data were collected using a structured questionnaire that included information on age, sex, disease duration, history of prior anti-VEGF injections, and presence of comorbidities. BCVA was measured using a Snellen chart in standardized low light conditions using a projected Snellen chart at 20 feet. BCVA was converted to a logarithm of the minimum angle of resolution (logMAR)^[^20^]^ for statistical analysis.

Spectral-Domain Optical Coherence Tomography (SD-OCT) reports from patient medical records were analyzed to assess central subfield retinal thickness (CSRT) and macular volume (MV). Imaging was performed using the Cirrus HD-OCT system (Model 500; Carl Zeiss Meditec). Only scans with a signal strength above 7/10 with fovea as the centermost point were included to ensure high-quality data. A 512 × 128 degree cube was used for OCT volume scan. CSRT was defined as the average thickness within the central 1-mm ring, while MV represented the total retinal volume within the central 3-mm and 6-mm rings.

Both anti-VEGF injections were administered under sterile conditions in an operating theater. Povidone-iodine 5% was applied to the eyes immediately before and after the injections, and postoperative care included 1 week of topical moxifloxacin 0.5%. Preoperative antibiotic eye drops were not utilized. The brolucizumab group included treatment-naïve patients (n = 3) and therapy-refractory cases (n = 7) who had not responded to prior anti-VEGF therapies. Brolucizumab was administered at a single dose of 6 mg (0.05 mL of 120 mg/mL solution) and visual acuity was assessed at follow-up visits. Ranibizumab was administered monthly as a single intravitreal injection at 05 mg (0.05 mL) for treatment-naïve patients. To assess and compare efficacy, follow-up evaluations were conducted at baseline (prior to treatment) and at 4, 8, and 12 weeks follow-up. These intervals allowed for a structured analysis of treatment outcomes between the two groups.

### Outcome measurements

The primary objective was to evaluate the changes in BCVA and OCT measurements (MV and CSRT) from baseline (prior to anti-VEGF treatment) to follow-ups at 4, 8, and 12 weeks post-treatment in patients treated with both brolucizumab and ranibizumab. The secondary objective was to compare the changes in BCVA and OCT measurements between brolucizumab and ranibizumab at the final follow-up (12th week).

Furthermore, the incidence of drug-related adverse events was reported from the medical records of the patients. Common adverse effects of brolucizumab included blurred vision, cataracts, conjunctival hemorrhage, eye pain, and vitreous floaters. For ranibizumab, typical adverse events included conjunctival hemorrhage, eye pain, vitreous floaters, retinal hemorrhage, vitreous detachment, intraocular inflammation, eye irritation, foreign body sensation, visual disturbances, increased lacrimation, blepharitis, ocular hyperemia, dry eyes, vitritis, and eye pruritus^[^15-27^]^.

### Data analysis

Descriptive statistics were completed relating to respondent’s characteristics which were expressed as frequencies and percentages for categorical variables and mean and standard deviations for continuous variables. Comparisons between baseline and the 12th-week follow-up were evaluated using paired t-tests, while comparisons between brolucizumab and ranibizumab at the final follow-up (12th week) were assessed using Student’s t-tests. Data analysis was performed using the Statistical Package for Social Sciences (SPSS) version 25, with a *P* value <0.05 considered statistically significant.

## Results

### Patient profile and demographics

Of the 10 patients who received brolucizumab, their ages ranged from 51 to 71 years, with an equal distribution of male and female participants (1:1). The duration of their disease varied between 2 and 6 years. Among these 10 patients, 2 had diabetes mellitus, while 8 had hypertension. Additionally, 7 individuals had previously been treated with anti-VEGF before switching to brolucizumab. The age of the 10 patients who were administered ranibizumab ranged from 42 to 73 years, and there was an equal representation of both male and female participants. The duration of their disease spanned from 0.4 to 5 years. Within this group, 2 patients had diabetes mellitus, while 6 had hypertension. Furthermore, none of the patients underwent prior treatment with other anti-VEGF agents before ranibizumab administration (Table 1).

**Table 1:** Case profile of patients undergoing treatment with Intravitreal brolucizumab and ranizumab (n = 20).

Case no	Duration of disease (years)	Presence of DM	Presence of HTN	Previous use of other anti-VEGF agents
*Cases administered brolucizuamb*				
Case 1	5	No	Yes	Yes
Case 2	3	No	Yes	Yes
Case 3	4	No	No	Yes
Case 4	2	No	No	Yes
Case 5	6	No	Yes	Yes
Case 6	4	No	Yes	Yes
Case 7	3	Yes	Yes	No
Case 8	3	No	Yes	No
Case 9	2	No	Yes	Yes
Case 10	2	Yes	Yes	No
***Cases administered ranizumab***				
Case 1	1	No	No	No
Case 2	0.5	Yes	Yes	No
Case 3	0.5	Yes	Yes	No
Case 4	0.4	No	Yes	No
Case 5	2	No	Yes	No
Case 6	5	No	Yes	No
Case 7	2	No	Yes	No
Case 8	0.5	No	No	No
Case 9	1	No	No	No
Case 10	0.5	No	Yes	No

BCVA = best-corrected visual acuity; CSRT = central subfield retinal thickness; MV = macular volume; DM = diabetes mellitus; HTN = hypertension.

### Changes in BCVA, CSRT, and MV in patients undergoing treatment with brolucizumab from baseline to 12th week follow-up visit

**Figure 1.**
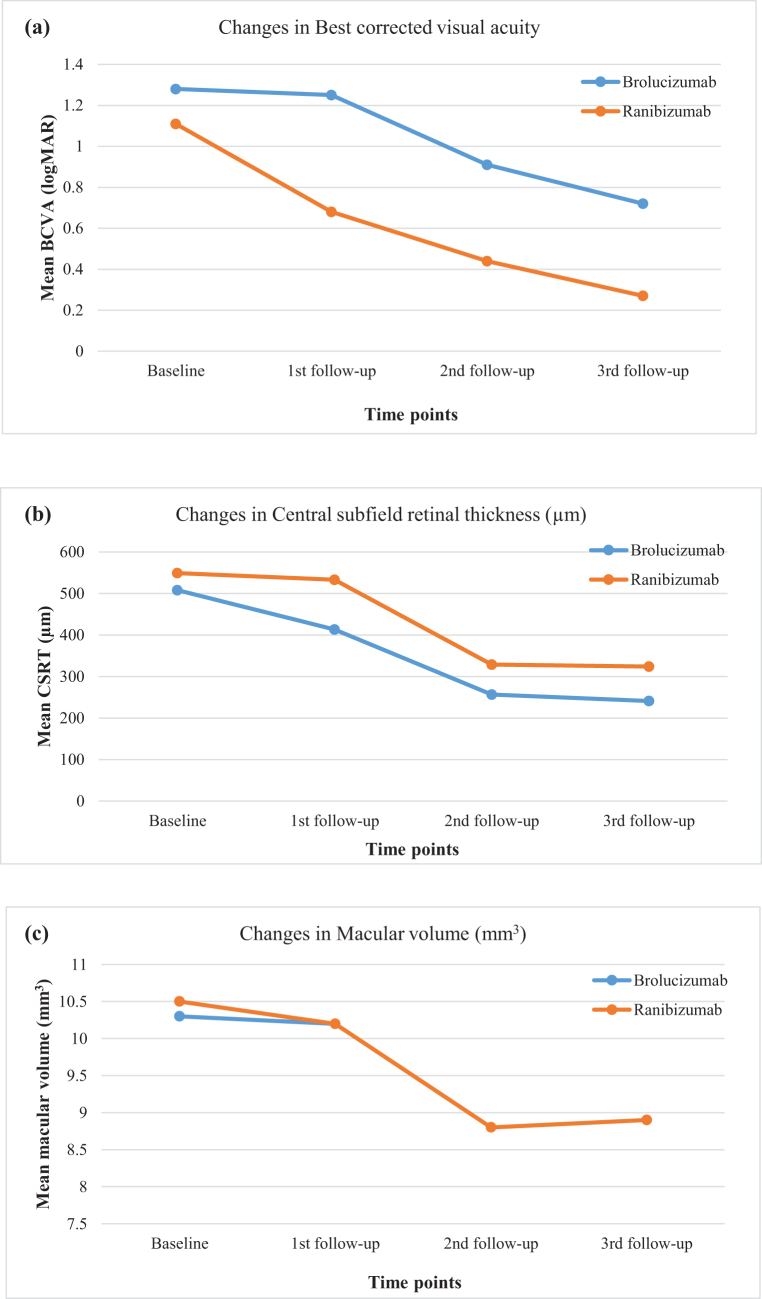
Changes in BCVA, CSRT, and MV from baseline to 12th week follow-up after brolucizumab and ranibizumab administration. (a) Changes in mean BCVA measured in logMAR. (b) Changes in mean CSRT. (c) Changes in mean MV.

The average BCVA significantly improved from the baseline (logMAR = 1.28) to 12th week follow-up (logMAR = 0.72) after initiation of brolucizumab treatment (*P* = 0.042). From baseline to 12th week of follow-up visit, the mean CSRT reduced from 508.5 ± 101.1 µm to 241 ± 40.4 µm and the MV reduced from 10.3 ± 1.0 mm^3^ to 8.2 ± 1.6 mm^3^. There was a significant difference in CSRT (*P* < 0.001) and MV (*P* = 0.002) from baseline to 12th week after administration of brolucizumab (Fig. 1).

### Changes in BCVA, CSRT, and MV in patients undergoing treatment with ranizumab from baseline to 12th week follow-up visit

The average BCVA significantly improved from the baseline (logMAR = 1.10) to 12th week follow-up (logMAR = 0.27) after initiation of ranibizumab treatment (*P* = 0.001). The mean MV significantly decreased from 10.5 mm^3^ at baseline to 8.9 mm^3^ at the 12th week of follow-up (*P* = 0.007). The CSRT over the follow-up period decreased significantly from baseline (549.3 µm) to the 12th week of follow-up (324.4 µm) (*P* < 0.05) (Fig. 1).

### Comparison of BCVA, CSRT, and MV changes in patients undergoing treatment with brolucizumab and ranibizumab at 12th week follow-up visit

At the 12th week follow-up, there were no statistically significant differences observed in BCVA, CSRT, and MV between patients who received brolucizumab and those who received ranibizumab (*P* < 0.05) (Table 2).

**Table 2:** Comparison of BCVA, CSRT, and MV changes in patients undergoing treatment with brolucizumab and ranibizumab at 12th-week follow-up (n = 20).

	Brolucizumab	Ranibizumab	*P* value
	n = 10	n = 10	
	Mean ± SD	Mean ± SD	
**Visual acuity**			
BCVA (logMAR)	0.72 ± 0.17	0.61 ± 0.50	0.055
**OCT measures**			
CSRT(/µm)	241 ± 40.4	324 ± 120.8	0.063
MV(/mm^3^)	8.2 ± 1.6	8.9 ± 1.9	0.397

BCVA = best-corrected visual acuity; CSRT = central subfield retinal thickness; MV = macular volume.

*P* value obtained by independent Student’s t-test.

Brolucizumab cases: paired t-test revealed a significant improvement in visual acuity (*P* < 0.05) over time in BCVA, CSRT, and MV.

Ranibizumab cases: paired t-test revealed a significant improvement in visual acuity (*P* < 0.05) over time in BCVA, CSRT, and MV.

Moreover, 8 out of 10 patients treated with brolucizumab did not have any adverse events during the study period, 1 patient reported conjunctival hemorrhage and 1 patient reported slight increase in intraocular pressure (IOP), whereas patient treated with ranibizumab did not show any adverse events all throughout the follow-up period.

## Discussion

Despite the progress made in treating AMD and DME, there are still unmet needs in managing these conditions. Patients often bear a substantial treatment burden, and the improvements in their vision and the reduction of fluid in the eye may not be sustained over the long term^[^12,24,25^]^. Brolucizumab presents itself as an anti-VEGF option for physicians, offering an extended duration of action and improved ability to dry retinal fluid compared to other anti-VEGFs^[^15,16,26^]^.

In our case series, we have demonstrated that brolucizumab is effective in improving and sustaining visual acuity up to week 12 in patients with both AMD and DME when administered as a single dose, with minimal adverse events. Our findings suggest that initiating intravitreal brolucizumab therapy is beneficial for treatment-naïve patients and those who have previously undergone multiple anti-VEGF injections without achieving satisfactory resolution of fluid in various anatomical compartments.

In our case series, 12 weeks post-injection of brolucizumab, measured visual acuity improved significantly. In the initial human trial known as the SEE study, it was observed that the time elapsed before a repeat injection was needed was extended by 30 days when using either 3 mg or 6 mg of brolucizumab compared to ranibizumab^[^27^]^. In the phase II OSPREY trial, approximately 50% of the eyes treated with brolucizumab maintained stable visual acuity when following a dosing schedule of every 12 weeks (q12w)^[^21^]^. Similarly, in the phase 3 HAWK and HARRIER trials, approximately 50% of patients were able to sustain the q12w dosing regimen for up to 48 weeks^[^15^]^ Among these eyes, approximately 75% continued successfully with the q12w injection schedule for as long as 96 weeks. This improvement could well be due to the increased penetrance of brolucizumab into retinal tissues owing to its smaller 26 kDa size compared to ranibizumab (48 kDa), aflibercept (115 kDa), and bevacizumab (149 kDa)^[^22,23^]^.

On the other hand, the results of our study also confirmed the efficacy of intravitreal brolucizumab in improving the anatomical outcomes, especially reducing CSRT and MV. While disparities in study design and analytical approaches make it challenging to draw a direct comparison, our results align with a recent real-world study conducted by Sharma *et al*. In their study involving 42 eyes of 42 patients with AMD, they also observed improvements in anatomical outcomes. This improvement occurred over an average observational period of 7.2 ± 3.6 weeks^[^28^]^. Additionally, in line with our research, Avaylon *et al* similarly identified favorable anatomical outcomes during the initial visit following a transition to brolucizumab treatment^[^29^]^.

A noteworthy concern with brolucizumab is its potential pro-inflammatory properties, first highlighted after its initial approval for treating nAMD^[^30-32^]^. In our case series, 8 out of 10 patients treated with brolucizumab experienced no adverse events, while 2 patients reported non-severe drug-related events, including conjunctival hemorrhage and elevated intraocular pressure (IOP). Importantly, no serious adverse events such as intraocular inflammation (IOI) or vasculitis were observed. Given that DME is inherently an inflammatory condition, there has been speculation that IOI might occur more frequently in these patient^[^33^]^. However, prior studies have shown that IOI rates are lower with the 6 mg dose of brolucizumab^[^15,34^]^.

However, the study found no statistically significant superiority of brolucizumab over ranibizumab in terms of visual acuity and OCT measures. This may be attributed to the small sample size, lack of randomization, and the relatively short follow-up period. Additionally, as the data were retrospectively collected from a database, standardized patient instructions could not be ensured, making the findings less comparable to those of controlled clinical trials.

Nevertheless, these findings have important clinical implications, particularly in resource-constrained settings where access to anti-VEGF therapy is limited. The results may also inform future clinical research and aid ophthalmologists in counseling patients regarding treatment options.

### Study limitations

Our study has certain limitations that should be acknowledged. Firstly, the retrospective design introduces a risk of selection bias. Additionally, the sample size was small, and the follow-up period was limited to 12 weeks, which may not capture long-term outcome and therefore limits generalizability. Our primary objective was to evaluate the initial treatment response to brolucizumab and ranibizumab in DME and AMD patients. The decision to use a 12-week follow-up period was intentional, as follow-up rates tended to drop significantly beyond this point, potentially leading to less reliable data. Factors such as treatment cost, geographic accessibility, and patient satisfaction with early improvements in vision and anatomy may have contributed to missed follow-up appointments later in the treatment course. In addition, addition of data about quality of life and visual functional score could be more useful to understand the beneficial effect of these drugs but cannot plotted due to lack of data. Nonetheless, these limitations highlight the need for larger, longer-term studies or randomized controlled trial to validate and expand upon our findings.

## Conclusion

In conclusion, our case series on brolucizumab in treatment-naïve and therapy-refractory DME and AMD patients demonstrated clinically significant improvements in visual acuity, with minimal adverse events observed over a 12-week period. A key advantage of brolucizumab is its reduced injection frequency, which may alleviate the treatment burden for patients, physicians, and the healthcare system in Bangladesh. However, larger-scale, randomized controlled trials are essential to validate these findings and further establish the therapeutic potential of brolucizumab in managing AMD and DME across diverse populations in Bangladesh.

## Data Availability

Data and materials pertaining to individual patients can be accessed upon request from the corresponding author.
